# The implication of metabolically active *Vibrio* spp. in the digestive tract of *Litopenaeus vannamei* for its post-larval development

**DOI:** 10.1038/s41598-020-68222-9

**Published:** 2020-07-10

**Authors:** Estefanía Garibay-Valdez, Luis Rafael Martínez-Córdova, Marco A. López-Torres, F. Javier Almendariz-Tapia, Marcel Martínez-Porchas, Kadiya Calderón

**Affiliations:** 1 Centro de Investigación en Alimentos y Desarrollo A.C (CIAD), Carretera a La Victoria S/N, CP. 83304 Hermosillo, Sonora Mexico; 2 Departamento de Investigaciones Científicas y Tecnológicas (DICTUS), Universidad de Sonora, Blvd. Luis Donaldo Colosio S/N, CP. 83000 Hermosillo, Sonora Mexico; 3 Departamento de Ingeniería Química y Metalurgia, Universidad de Sonora, Blvd. Luis Donaldo Colosio S/N, CP. 83000 Hermosillo, Sonora Mexico

**Keywords:** Ecology, Microbiology

## Abstract

This work aimed to evaluate the link between the occurrence/abundance of *Vibrio* populations and bacterial composition in shrimp’s intestine (*Litopenaeus vannamei*) during post-larval ontogenetic development and in its culture water, and the correlation of these with environmental parameters. The total and metabolically active populations of *Vibrio* in the digestive tract of shrimp during its post-larval development were analysed using quantitative PCR (qPCR) and reverse transcription qPCR targeting the 16S rRNA gene sequence. A lab-scale shrimp bioassay was performed for 80 days in a recirculating aquarium under strictly controlled conditions. The results indicate that the *Vibrio* population from shrimp’s gut is associated with its developmental stage and the environment. Multivariate analyses revealed that the presence of *Vibrio* spp. drove the studied system, but their metabolically active performance was related to earlier developmental stages in an aqueous environment. Also, the samples taken from water of culture units to compare the influence of the aquatic environment on the intestinal microbial community during shrimp’s ontogenetic development showed significant differences. Finally, our results revealed that *Vibrio* is an important member of shrimp’s gut microbiota; however, its metabolic activity seems to be highly regulated, possibly by the host and by the rest of the microbiota.

## Introduction

Shrimp aquaculture plays an important role in the world’s economy. In the western hemisphere, Mexico is the sixth-largest producer of shrimp by aquaculture and, specifically, 37% of this production is provided by the State of Sonora, where the Pacific white shrimp, *Litopenaeus vannamei* is one of the most exported species around the world for human consumption due to its edible protein and nutrients^1,2^. In the last decade, the high demand from the aquaculture market, the quickly growing rate of shrimp production, and intensive farming practices have made these shrimp susceptible to disease and mortality, causing spoiled cultures and irreparable economic losses for the aquaculture industry^2–4^.

One of the principal factors threatening shrimp production is related to biotic factors, which encompass virus and bacterial infections, and abiotic factors which are mostly linked to intensive farming practices, triggering the accumulation of carbonaceous, nitrogenous, and phosphorous species in farming ponds^1,3^. Shrimp diseases caused by opportunistic bacteria such as bacteria and viruses are the major problems that can lead to considerable losses in the shrimp farm industry. The main shrimp diseases are caused by bacterial pathogens mainly from *Vibrio* species such as *V. harveyi*, *V. alginolyticus*, *V. campbellii*, and *V. parahaemolyticus*, causing necrosis, slow growth, anorexia, and mortality during the post-larval development^5^. In this regard *V. parahaemolyticus* strains constitute the etiological agent of the most relevant bacterial disease recorded in shrimp to this day, which has severely disrupted the shrimp industry containing a unique virulence plasmid with genes encoded for delta endotoxins (pir A and pir B), similar to the *Photorhabdus* insect-related (Pir) toxin, and causing mortality in the infected organisms^2,6^. However, non-pathogenic species such as *V. alginolyticus* and *V. gazogenes* inhibit the growth of other pathogenic *Vibrio.* For instance, *V. alginolyticus* has shown antagonistic activity and *V. gazogenes* has potential as a probiotic for the control of *Vibrio* infections after oral application in the Pacific white shrimp, *L. vannamei*^7^. In other invertebrates like copepods, it is established that they are capable to discriminate between different *Vibrio* species, presenting different transcriptional immune responses that may favour the acquisition of bacterial symbionts from the environment such as non-pathogenic *Vibrio*^8^. In this sense, Wendling and Wegner^9^ mentioned that the Pacific oyster (*Crassostrea gigas*) presents a fast evolutionary response and a genetic resistance mechanism against *Vibrio* virulence factors. Besides, the expression of *Vibrio* virulence factors depends on the resident microbiota of the host; therefore, a *Vibrio* disease breaks out synergistically with the microbiome, which may include other non-virulent *Vibrio*.

Research on *Vibrio* disease should focus on the biotic interactions between the environment, the host, and the pathogenic and non-pathogenic fractions of microbial communities^10^. For instance, *Vibrio* pathogens can interact with symbiotic communities and the host microbiota, to invade the host’s intestine manipulating its biomechanics to redefine gut communities. On the whole, understanding the ecological processes implicated in diseases is complicated due to the high diversity of the *Vibrio* populations, and because the biotic interactions within and between microbial communities modify the disease expression on different levels^10–12^.

In the same way, the microbial communities could provide guest protection against pathogenic infections because some microorganisms produce antimicrobial complexes^13,14^. Also, the gut microbiota of shrimp has become more relevant due to our knowledge of its biological interactions. The microbial colonization process in the gut can be driven by populations that set the optimal conditions for colony formation, where other microbes become attached to the intestinal tissue and create strong interactions with the microbial consortium formed while displacing pathogens or undesirable microbes that could affect the host and efficient intestinal function^3,13–16^. Therefore, studying aspects related to the behaviour of the gut microbiota may contribute to our understanding of these complex communities. In addition, the microbiota varies during the development of most animals, according to their requirements and other underlying factors; thus, this response is also expected in shrimp, but what changes or how these changes occur remains unknown. Information about the *Vibrio* dynamics in shrimp gut would contribute to understand this complex relationship between *Vibrio* and penaeid shrimp.

Despite efforts to improve shrimp production, there are only a few studies based on microbial ecology that link the occurrence of relevant pathogenic microbial groups and the metabolic activities of these groups, causing dysbiosis in white shrimp, which is related to their ontogenetic development. In the present study, the total population and the metabolically active population of *Vibrio* sp. were evaluated in a lab-scale system with Real-time polymerase chain reaction (qPCR) and reverse transcription qPCR (RT-qPCR), targeting the 16S rRNA gene sequence. The link between the occurrence/abundance of target populations and bacterial composition during post-larval ontogenetic development and their correlation with environmental parameters and nutrients were investigated by multivariable analyses [principal component analysis (PCA)] in the intestines and culturing water.

## Results

Biometry and productive response analyses of *L. vannamei* were evaluated, registering significant differences associated with the developmental stage; for example, between the control (day 0), 20–40 days of development, and 60–80 days of development (Table 1). Moreover, the productive response of shrimp was as expected, with higher growth performance from days 1 to 40 and a stationary phase achieved at days 60–80. The specific growth rate (SGR) was estimated to explain the rate of increase of the *L. vannamei* population from one sampling day to another, with each interval belonging to a developmental stage. There were significant differences in the SGR between all post-larval stages. From the first stage, the SGR was 5.60% day^–1^, followed by the second stage with 4.24% day^–1^. At stages III and IV, the SGR of shrimp decreased to 1.36% and 0.33% day^–1^ respectively, with a global SGR of 2.88% day^−1^ for the entire bioassay (Table 1). No significant differences were found between the developmental stages in regards to temperature (24.8–25.4 °C), salinity (35.36–36.25%), DO (6.4–6.9 mg L^–1^), and pH (8.1–8.4), demonstrating that the entire experiment was well controlled on a lab-scale (Table 2). Regarding water quality, significant differences were recorded for some parameters (Table 3). High concentrations of NH_3_–NH_4_ were observed at 20 days of development (5.34 mg L^–1^), while low values were recorded at days 40 and 60 (0.93 and 1.2 mg L^–1^, respectively), and statistically similar but lower values at days 0 and 80 were recorded (0.39 mg L^–1^). The concentrations of nitrite (NO_2_–N) and nitrate (NO_3_–N) were higher at day 60 (0.27 mg L^–1^ and 10.2 mg L^–1^, respectively), followed by day 40 (0.11 mg L^–1^ and 6.50 mg L^–1^, respectively), compared with the rest of the sampled days (0, 20, and 80 days). The highest concentrations of phosphate were registered at days 20, 40, and 60, all with values ranging within 1.6–1.98 mg L^–1^.

**Table 1:** Biometrics data during shrimp development and specific growth rates (SGRs) of *Litopenaeus vannamei* during the experimental period.

Development stage	Days of development	Initial BL (cm)	Final BL (cm)	Initial weight (g)	Final weight (g)	SGR (%day-1)
Control	0	3.2 ± 0.1^(d)^	–	0.3 ± 0.1^(d)^	–	–
I	1–20	4.40 ± 0.71^(c)^	6.57 ± 0.80	0.75 ± 0.34^(c)^	2.32 ± 0.83	5.60^(a)^
II	21–40	6.57 ± 0.80^(b)^	8.59 ± 1.00	2.32 ± 0.83^(b)^	5.41 ± 1.87	4.24^(b)^
III	41–60	8.59 ± 1.00^(a)^	9.37 ± 1.11	5.41 ± 1.87^(a)^	7.15 ± 2.5	1.36^(c)^
IV	61–80	9.37 ± 1.11^(a)^	9.68 ± 0.93	7.15 ± 2.5^(a)^	7.59 ± 2.09	0.33^(d)^
					**Global SGR**	**2.88**

BL corresponds to biometric length. Initial BL and weight refer to the first day of each stage and Final BL and weight refer to the final day of each stage. Values shown are averages ± standard deviation SD. LSD, least significant difference (Student’s test, *p* < 0.05). *Significant differences between stages I, II, and III, corresponding to 0–20, 20–40, and 40–60 days of shrimp development, respectively (*p* < 0.05).

Different letters indicate significant differences among development stages at a 95% confidence level.

**Table 2:** Physical–chemical parameters of water monitored during the bioassay.

Development stage	Days of development	Temperature (°C)	DO (mg L^−1^)	Salinity (%)	pH
I	1–20	25.4 ± 0.05	6.7 ± 0.01	36.25 ± 0.10	8.4 ± 0.01
II	21–40	25.0 ± 0.07	6.4 ± 0.23	35.57 ± 0.07	8.1 ± 0.01
III	41–60	24.8 ± 0.09	6.8 ± 0.02	35.49 ± 0.07	8.2 ± 0.01
IV	61–80	24.8 ± 0.16	6.9 ± 0.02	35.36 ± 0.03	8.1 ± 0.01

*Significant differences (*p* < 0.05).

**Table 3:** Mean ± SD of water quality parameters of nitrite (NO_2_–N), nitrate (NO_3_–N), ammonia (NH_3_–NH_4_), and phosphate (P–PO_4_) during shrimp culture days as Temperature.

Sample	NH_3_–NH_4_ (mg L^−1^)	NO_2_–N (mg L^−1^)	NO_3_–N (mg L^−1^)	P–PO_4_ (mg L^−1^)	Temperature (°C)	Salinity (%)	DO (mg L^−1^)	pH
W0	0.39 ± 0.01^(c)^	0.01 ± 0.00^(c)^	0.0 ± 0^(c)^	0.17 ± 0.03^(b)^	25.2 ± 0.3	37.9 ± 0.7	6.87 ± 0.05	8.3 ± 0.0
W20	5.34 ± 0.16^(a)^	0.04 ± 0.00^(c)^	0.93 ± 0.49^(c)^	1.98 ± 0.20^(a)^	25.1 ± 0.6	35.4 ± 0.3	6.45 ± 0.27	8.4 ± 0.1
W40	0.93 ± 0.09^(b)^	0.11 ± 0.00^(b)^	6.50 ± 1.67^(b)^	1.66 ± 0.08^(a)^	24.7 ± 0.7	35.2 ± 0.1	6.57 ± 0.25	8.1 ± 0.1
W60	1.20 ± 0.03^(b)^	0.27 ± 0.05^(a)^	10.2 ± 1.30^(a)^	1.88 ± 0.30^(a)^	24.3 ± 1.1	35.3 ± 0.4	7.07 ± 0.13	8.1 ± 0.1
W80	0.39 ± 0.01^(c)^	0.01 ± 0.00^(c)^	0.0 ± 0^(c)^	0.17 ± 0.03^(b)^	24.9 ± 1.5	35.1 ± 0.0	7.07 ± 0.13	8.0 ± 0.0

Different letters in the same column indicate significant differences among treatments at a 95% confidence level.

**Figure 1:**
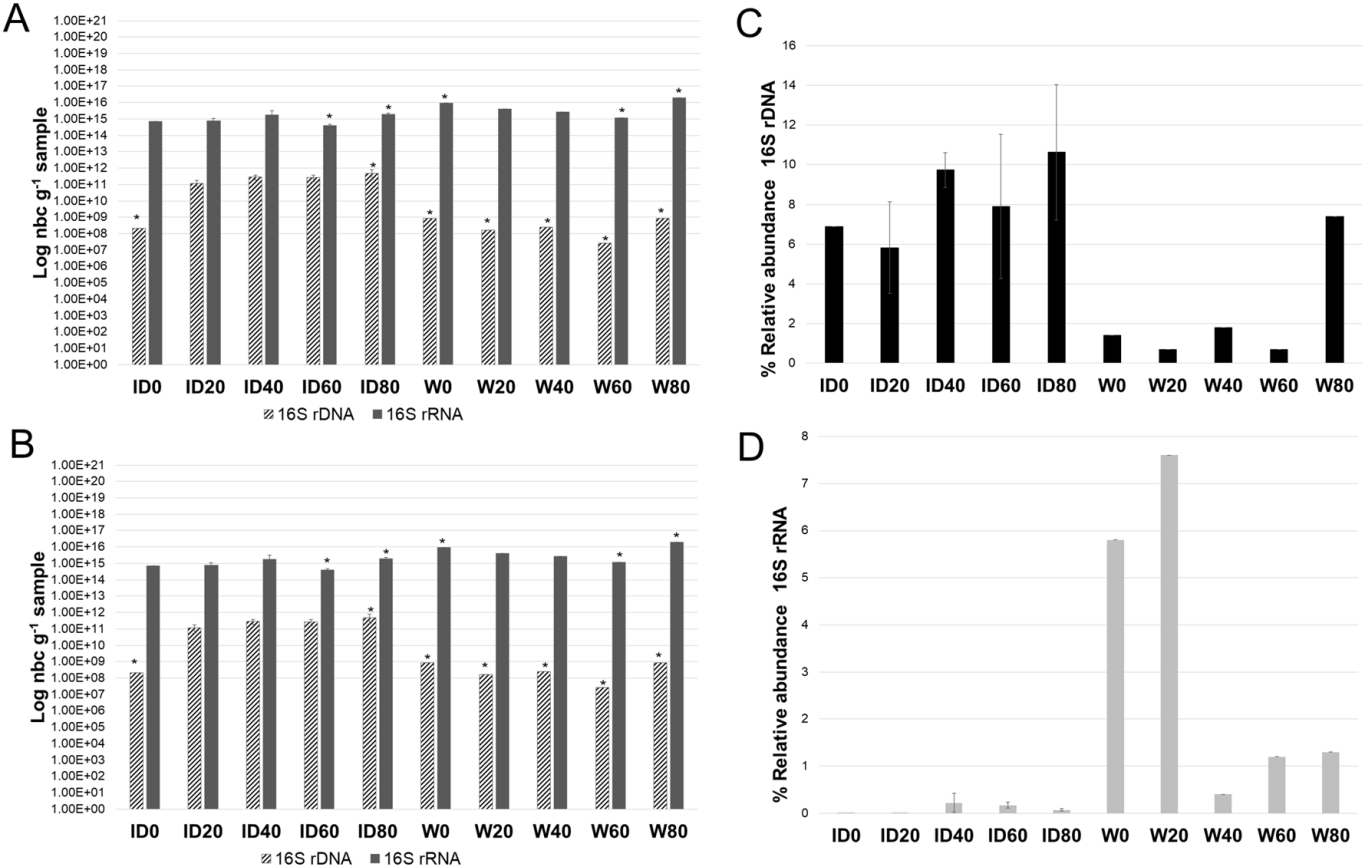
Logarithm of the numbers of copies (nbc) of 16S rDNA genes and 16S rDNA of bacteria (**A**) per gram of intestine (ID) and water (W) sampled, and the logarithm of the numbers of copies (nbc) of 16S rDNA and 16S rRNA of *Vibrio* (**B**) per gram sampled at intestine (ID) and water (W). Bars marked with the asterisk are significantly different according to the Kruskal–Wallis test (*p* < 0.05). Average relative abundances of *Vibrio* sp. 16S rDNA (**C**) and 16S rRNA (**D**) copies, expressed as the percentage of 16S rDNA copies of bacteria, in shrimp intestine (ID) and water (W). Bars marked with the asterisk are significantly different, according to the Kruskal–Wallis test (*p* < 0.05).

Regarding qPCR and RT-qPCR analyses, all average abundances were expressed as a logarithmic scale of the number of copies of 16S rDNA or 16S rRNA of total bacteria and *Vibrio* spp., quantified per gram of sample (water or intestine, respectively), corresponding to 0, 20, 40, 60, and 80 days of development (Fig. 1A, B). The Kruskal–Wallis and Conover-Iman tests showed that the differences among the quantifications of each target group, based on either DNA or RNA were always significant, with the metabolically active populations being at least four orders of magnitude higher, especially for total bacteria (Fig. 1). Significant differences were also observed between the different origins of the samples, where values of water samples were often higher than values of intestine samples for both target groups and only occasionally for the stage of development of the shrimp.

The results of the average relative abundances of *Vibrio* spp. compared to the total bacteria showed significant differences based on the environmental group studied, such as the intestine or water, and significant differences were observed for both groups when comparing the occurrence (16S rDNA copies) and the metabolically active specimens (16S rRNA copies) (Fig. 1). The occurrence of *Vibrio* 16S rDNA copies in water ranged from 0.7 to 7.4% (Fig. 1C). Simultaneously, the relative abundance of the metabolically active *Vibrio* community ranged from 0.4 to 7.6% (Fig. 1D). Additionally, at the beginning of the bioassay (W0), the *Vibrio* community presented higher metabolic activity compared to the total relative abundance (16S rDNA). At W80, the relative abundance of *Vibrio* was higher than the metabolically active abundance, which is related to the total bacteria (Fig. 1C, D). Meanwhile, the relative abundance of the entire *Vibrio* community in the intestines of shrimp was higher (5.83–10.63%) than that found in water samples (0.4–7.6%). In addition, the relative abundance of metabolically active *Vibrio* (16S rRNA) among total bacteria was mostly higher in water than in shrimp intestines throughout the bioassay.

**Figure 2:**
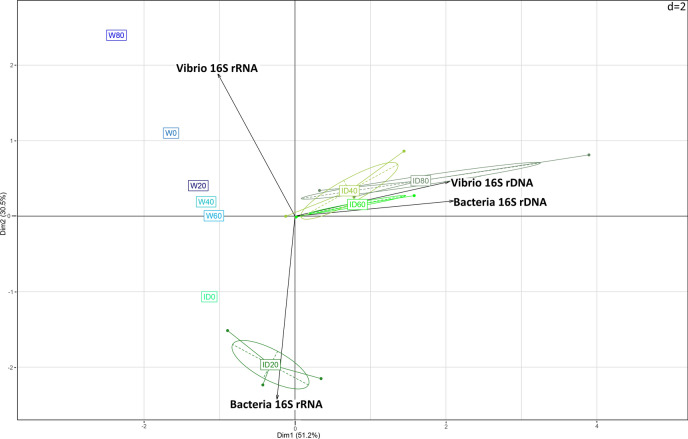
Principal component analysis (PCA) based on Euclidean distances illustrating the component of four sets of biotic data derived from the quantifications by qPCR/RT-qPCR in water and intestine samples. The number of copies per g of sample of 16S rRNA genes (16S rDNA) and 16S rRNA was used to explain the abundance of total bacteria and *Vibrio*.

To analyse the biological system related to the total abundance and the metabolically active expression of total bacteria and the *Vibrio* spp. communities, PCA based on Euclidean distances was performed (Fig. 2), where the first axis explained 51.2% of the total variability in the entire biological system. The second axis also explained 30.5% of the variability, meaning that 81.7% of the total variability of the studied system was explained, considering the four sets of biotic data derived from the quantifications by qPCR/RT-qPCR in water and intestine samples. The entire system was first driven by the presence of total bacteria (bacteria 16S rDNA), which theoretically includes all bacterial taxa thriving in the system (including the *Vibrio* population), followed by the presence of the *Vibrio* community (*Vibrio* 16S rDNA). In addition, different clusters were clearly formed, depending on the tissue evaluated [water (blue) or intestine (green)]. Herein, one isolated cluster was formed, corresponding to the intestines of post-larval organisms from the farm (ID0), and another neighbouring cluster was also formed, corresponding to samples of the first stage of post-larval development, corresponding to 20 days (ID20) of trial, which was strongly correlated to the metabolically active performance of total bacteria (bacteria 16S rRNA). Interestingly, the metabolically active abundance of total bacteria (16S rRNA) was negatively correlated with the metabolically active abundance of the *Vibrio* population (*Vibrio* 16S rRNA), which was, at the same time, the biotic parameter that managed the studied water environment. Additionally, clusters derived from intestine samples from D40, D60, and D80 formed a single cluster, suggesting a similar microbiota pattern during late post-larval development (juvenile–adult) compared to early stages from D0 and D20 (post-larvae).

**Figure 3:**
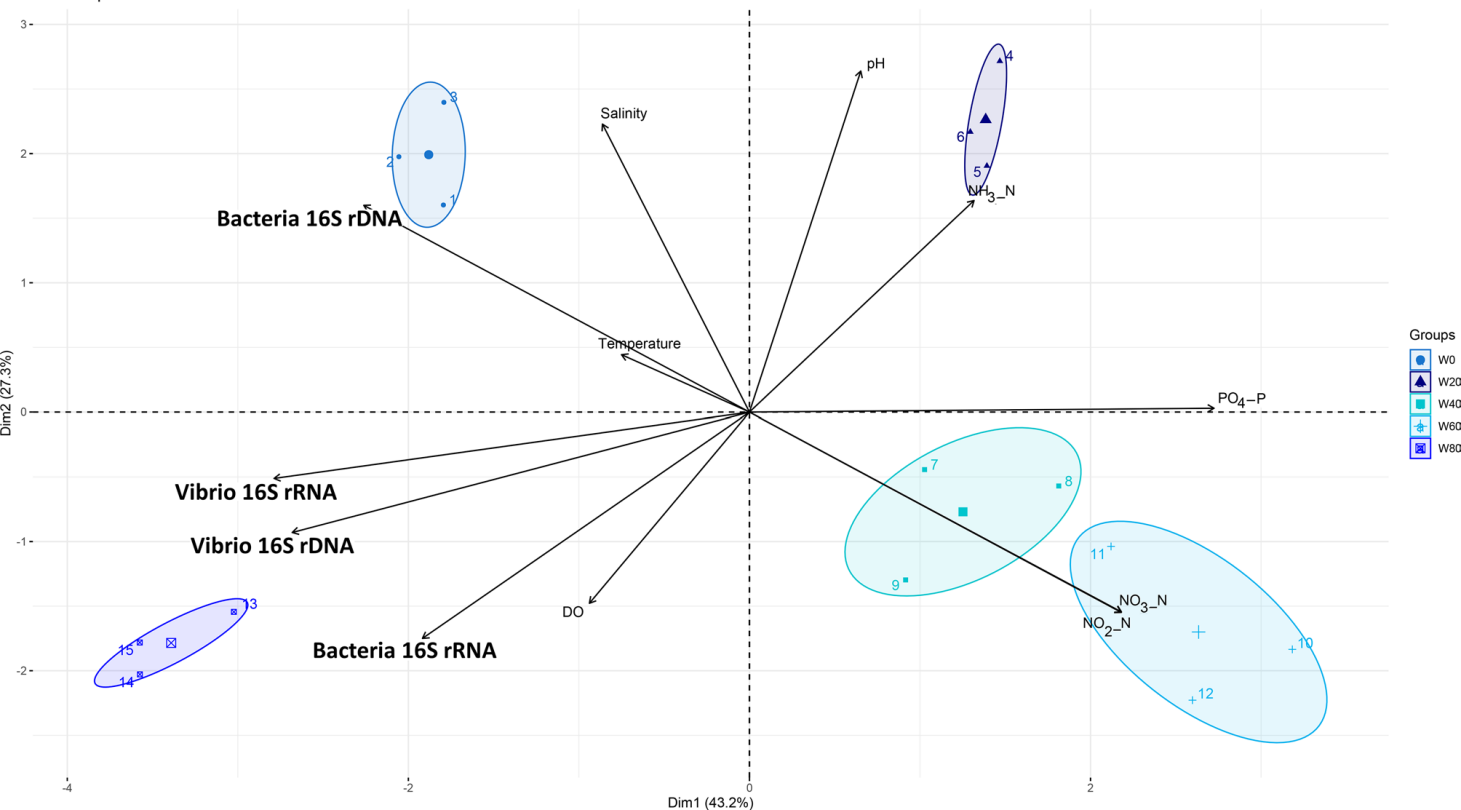
PCA based on Euclidean distances to analyse the influence of abiotic variables, such as NO_2_–N, NH_3_–NH_4_, NO_3_–N, P–PO_4_, dissolved oxygen (DO), salinity, pH, and temperature, over the total bacteria and *Vibrio* from water samples, based on the number of copies of 16S rRNA genes (16S rDNA) and 16S rRNA*.*

Furthermore, to evaluate the entire system, considering the four sets of biotic parameters and the influence of abiotic parameters in an aqueous environment, another PCA was performed (Fig. 3). As a result, 70.5% of the total variability of the biological system was explained, resulting from 43.2% in the first axis and 27.3% from the second axis. Different clusters were clearly formed, depending on the developmental stage of the white shrimp. The results revealed that for the water environment abiotic factors, such as nutrient accumulation (P–PO_4_), are key players that manage bacterial performance. A separate cluster of samples from W0 was positively correlated with the total bacterial occurrence, where temperature and salinity played an important role. Control samples (W0) were negatively correlated with W40 and W60, which were strongly and positively correlated with nitrate and nitrite accumulation in water. The abundances of the total and metabolically active *Vibrio* community displayed similar trends in response, where samples from W80 were positively correlated according to this biotic set of data and according to the metabolically active performance of total bacteria, where DO played an important role by its positive correlation with W80. Finally, samples from early developmental stages (W20) were positively correlated with ammonia (NH_3_–NH_4_) accumulation in water, as well as with pH.

## Discussion

To evaluate the growth of the white shrimp, biometric analyses were performed, demonstrating significant differences based on the developmental stage of the shrimp, where two main groups were formed. The first stage encompassed organisms from days 1 to 40, where their growth was notorious, and the second stage encompassed organisms that were still in the growth process but at a much lesser rate, registering no significant differences between days 60 and 80. The first group corresponded to shrimp during the first post-larval developmental stage, while the second group corresponded to shrimp on the 60th day of development, when shrimp were considered juveniles. Therefore, the shrimp’s nutritional requirements change according to its development and biological behaviour. Previous reports demonstrated that the gut enzymatic profile differs between each stage in order to achieve better nutrient absorption^17^. In addition, Xiong et al.^18^ showed that the shrimp growth rate is linked to its microbiota since organisms of the same age and culture time may have differences in their growth rate, and they present similarities between the gut community assembly according to their growth. In the present study, there was a significantly higher growth rate during the post-larval phase compared to the juvenile–adult phase. In this work, when the shrimp reached a mature stage, asymptotic growth rates were registered^19^.

Based on qPCR and RT-qPCR analyses, the abundance of the total bacterial community (16S rDNA and 16S rRNA) in the shrimp intestine was demonstrated to be stable from day 20 and onwards. Before that, samples from the control (ID0) were significantly lower than other culture days. Despite this, some factors including disease, nutrition, and environment, can regulate the gut microbiota. Our results suggest that a colonisation process occurs during the post-larval phase, reaching stability during the juvenile to adult phase, reducing the risk of dysbiosis^20–22^. However, our results demonstrated that the presence of metabolically active bacteria decreased at culture days 40 and 60, and the amount of this bacteria increased on day 80, with no significant differences concerning days 0 and 20.

The total abundance of the bacterial community in water samples was constant during the bioassay, except for the control (day 0) where the abundance was significantly higher. However, the abundance of metabolically active bacteria was the highest on day 80, and the abundance was significantly higher than days 0, 20, and 60.

Previous studies have revealed that both shrimp and its culture water share the same bacterial composition, meaning that shrimp can have similar bacterial assemblages as their culturing media^23,24^. Also, species richness tends to be higher in water samples than in gut shrimp microbiota^18^, probably because bacteria harboured by shrimp are subjected to bottleneck conditions, favouring particular specimens.

In this study, by exploring the dynamics of the occurrence of the *Vibrio* population, it was found that throughout the bioassay the abundance levels were significantly lower in the intestine on the first culture day (ID0). Conversely, the metabolically active *Vibrio* population did not show significant differences in the shrimp intestine during the post-larvae to adult period. However, the total *Vibrio* community in water tended to decrease on days 20, 40, and 60, and it increased again on day 80. Notably, metabolically active *Vibrio* was significantly higher in water than metabolically active *Vibrio* in shrimp intestines, despite the relative abundance of the total *Vibrio* (16S rDNA) community, which was higher in shrimp intestines. Notably, it is important to highlight that our study was performed with healthy shrimp, where the abundance levels in the total *Vibrio* community and the metabolically active community were similar.

Previous authors have reported that increases in the *Vibrio* community or specific bacterial variations in shrimp intestines or water are considered indicators of disease^24,25^. However, their presence could be associated with their metabolically active performance, but this is not determined only by their occurrence. Gainza et al.^26^ demonstrated that the proportion of the *Vibrio* population in shrimp gut microbiota based on V2–V3 amplicon sequencing, was higher during the nursery stage (2.02%) than in the harvest stage (0.64%), revealing that harvest stage microbiota is more diverse than nursery microbiota. Huang et al.^27^ argued that the microbiota of culture ponds (water or sediment) differs from shrimp gut microbiota. Also, the community abundance in shrimp intestines is not related to the surrounding environment (water), because the environmental microbiota is exposed to higher fluctuations of nutrients and other influential factors.

There were no significant differences in the average relative abundances of the total *Vibrio* community (16S rDNA) among all bacterial populations in shrimp intestines across the bioassay related to the developmental stages. Conversely, the average relative abundance of the 16S rRNA number copy of the *Vibrio* population among all bacterial communities in shrimp intestines was lower than 1%. Our study demonstrated that despite the total *Vibrio* population being higher in healthy shrimp intestines, this community was not metabolically active. This indicates that the metabolic activity is the main factor causing disease through intestinal dysbiosis due the fact that gut microbiota has essential roles in host development, acting as a natural barrier against pathogens^18,28^. Species from the *Vibrio* genus are known to be pathogenic to shrimp; although *Vibrio* bacteria are natural inhabitants in shrimp intestines and could be controllable under optimum culture conditions^29,30^.

Our results demonstrated that *Vibrio* was highly metabolically active in the water environment at days 0 and 20 but not in the intestine, where only its occurrence was detected. This indicates that the *Vibrio* population plays a key role as part of the whole bacterial community structure during the first days of shrimp cultivation corresponding to post-larval stages (0–20 days). In this sense, aquatic organisms have a close relationship with the microbiota present in the water environment due to the exchange of the intestinal microbiota and the microbiota occurring in water, meaning that shrimp and their microbiota were working together as a holobiont. However, the planktonic microbial population is not part of this holobiont, but they share the same environment and shrimp gut is exposed to the aquatic environment representing an important portal of entry for microorganisms. This complete relationship may be considered as an hologenome^31^. Like many other microorganisms, *Vibrio* is naturally in marine and estuarine environments, both as free-floating cells and attached to chitinous surfaces; however, it is well known that not all *Vibrio* strains are pathogenic. In a previous study developed by our research group^32^, we explored the bacterial composition diversity of the intestinal microbiota of the white shrimp and their structure during the ontogenetic development using next-generation sequencing analysis, and interestingly it was observed that the *Vibrio* population does not colonize at all stages of development, but only during the post-larval stage (0 and 20) in contrast with juvenile stage (Fig. S1). This could be explained due to the high levels of metabolically active abundance of *Vibrio* at the post-larval stages in water environment. In addition, the total diversity is the total number of species presented in a niche^33^; thus, the *Vibrio* population behaviour presented in the shrimp gut can be explained if we consider that the community structure are assembled by different species that conform the whole microbiome.

Although, in water at W80, the presence of metabolically active *Vibrio* increased, presenting similar activity to that recorded at day 60 (W60), suggesting low opportunity for infection compared to the metabolically active community on the initial culture days. Thus, in a case where a *Vibrio* infection could develop in a shrimp culture at a farm scale, it could probably be associated with the loss of culture due to its high metabolic activity in water. Thus, the importance of tracking abiotic factors, such as water quality parameters and nutrients, is fundamental in order to avoid vibriosis as a result of its metabolic activity. In particular, water temperature, salinity, and eutrophication level can lead to shrimp stress and, eventually, reduce bacterial host selection and shrimp disease^34,35^. The results of this study demonstrate that the total relative abundance of *Vibrio* spp. in shrimp intestines is higher than its metabolic activity. This is in agreement with previous studies, where innate immune system activation of shrimp, such as *Marsopenaeus japonicus* and *L. vannamei*, was evaluated in response to *V. alginolyticus* and *V. parahaemolyticus* infections, demonstrating that they could modulate host signalling proteins and activate their defence system in the presence of potentially pathogenic microorganisms, activating genes that have activities related to immune response mechanisms, such as prophenoloxidase and superoxide dismutase activities, and apoptosis in the hepatopancreas tissue^36,37^ as well as in the intestinal epithelium^38^. Therefore, a selective biological pressure exerted by the host on the metabolic activity of this bacteria could be occurring, while stimulating the shrimp's immune system by functioning as a natural probiotic necessary for the life cycle of the host.

## Conclusions

This study provides an overview of the interactions between the intestinal microbiota of the white shrimp and its environment, emphasising the ratio and correlation between the total and metabolically active *Vibrio*. Both the total and active populations of *Vibrio* indicated that there is colonisation of this bacterium in the white shrimp intestine throughout its post-larval development, but with low metabolic activity. Therefore, *Vibrio* is part of the intestinal microbiota of the shrimp, and it could be playing dualistic role, involved in a symbiotic relationship between the organism and the bacterial community structure of the intestinal microbiota, while acting as an opportunistic pathogen under certain circumstances. Our results suggest that the rest of the microbiota could probably regulate *Vibrio* activity, possibly establishing a coexistence mechanism that does not affect the host, providing conditions for the thrive of the microbial community. In contrast, the abundance of the metabolically active *Vibrio* population was greater in crop water, despite its low total abundance. Finally, our results demonstrate that despite *Vibrio* can be a common and inclusively dominant member of the white shrimp microbiota, its activity could be regulated by the rest of the microbiota and by the shrimp.

## Materials and methods

### Sampling of organisms and bioassay experiments

Shrimp post-larvae (PL5) of *Litopenaeus vannamei* were collected from the aquaculture farm Parque acuícola Cruz de Piedra, Guaymas, Sonora, Mexico (27° 51′ 05.9″ N 110° 31′ 57.0″ W). Afterward, post-larval shrimp were transported in aerated tanks with the same pond water as the farm to the Departamento de Investigaciones Científicas y Tecnológicas de la Universidad de Sonora (DICTUS), where a lab-scale system was previously tested and used. The bioassay was conducted for 80 days with healthy shrimp, each weighing 0.5 ± 0.1 g, and post-larvae were randomly distributed in the lab-scale system. The system consisted of nine 80-L culture units linked to a recirculation aquaculture system (RAS), and sterile seawater was used to fill the units to an operating volume of 60 L. Influent seawater was filtrated and flowed through a UV lamp for sterilization, and the seawater was equally dispensed to all culture units, as depicted in the supplementary material (Fig. S2). The culture units were maintained under similar indoor conditions with artificial aeration (2000F heat bonded silica; pore size, 140 µm), and the salinity was maintained at around 35‰ with the addition of sterile freshwater (MilliQ grade, Millipore) to avoid the incorporation of outside bacteria and to compensate evaporation. Finally, the effluent generated by the system flowed through a biofilter containing nitrifying bacteria to control toxic nitrogen compounds in the recirculation system (Fig. S2). The unconsumed feed, feces, moults, and dead organisms (if any) were removed daily.

The salinity, dissolved oxygen (DO), pH, and temperature were measured twice per day (07:00 and 18:00 h) using a YSI multiprobe system 556 (YSI Incorporated).

The bioassay started with PL5 on day 0. At this point, 40 organisms were randomly introduced into each culture unit, and the experiment lasted 80 days. Throughout the experiment, shrimp were fed twice a day at a rate of 4% wet biomass day^–1^ using feeding trays with the same formulated feed consisting of commercial grow-out pelletized feed with 25% crude protein, 5% lipids, and 4% fiber.

### Water quality and productive response

The water quality was monitored daily throughout the bioassay, and samples were collected weekly from each culture unit using sterile falcon tubes by filtering the water through 0.45 μm membranes (Millipore). Nitrite (NO_2_–N), nitrate (NO_3_–N), ammonia (NH_3_–NH_4_), and phosphate (P–PO_4_) concentrations were measured using commercial Hanna reagent kits HI 93707-01, HI 93728-01, HI 93700-01, and HI 93717-01, respectively (Hanna Instruments, Romania).

Biometry analyses were performed at the four different developmental stages, denominated as I, II, III, and IV, corresponding to 0–20, 20–40, 40–60, and 60–80 culture days, respectively, and the productive response was calculated^15^.

### Collection of intestine and water samples and DNA and RNA extraction

To discard the transitory microbiota, the shrimp were fasted for 6 h before sampling. Once the intestines were empty, these were dissected on the corresponding dates using a sterile dissection kit, placed in sterile cryogenic tubes, and stored at – 80 °C until nucleic acid extraction. Each sample date belongs to an experimental unit with three culture tanks. The intestine samples from culture tanks were pooled and considered as a replicate, giving three replicates per sampling time. At the same sampling points, 1 L samples of water (W) from each culture unit corresponding to an experimental replicate were collected and pooled for filtration through 0.22 µm sterile filters of mixed cellulose ester membrane (Whatman, Sigma, St Louis, USA) and placed in sterile, 50 mL falcon tubes for storage at – 80 °C until nucleic acid extraction.

Total DNA and RNA were extracted and purified from the membrane filters previously used to filter seawater samples and from intestines that were also previously sampled, both of them with the FastDNA Spin Kit for Soil^15^, and the FastRNA Pro Blue Kit (MP-Bio, Santa Ana, CA, USA) in combination with mechanical lysis using the FastPrep Systems (MP-BIO, Santa Ana, CA, USA). The obtained RNA samples were digested according to the TURBO DNA protocol (Ambion, Life Technologies Corporation, Carlsbad, CA, USA) and EDTA to stop the DNase activity and to ensure that any DNA residuals were presented. Finally, samples were purified according to the RNA Cleanup protocol from the RNeasy Mini Kit (Qiagen, Hamburg, Germany). The quality and concentration of nucleic acids were tested as previously described Maza-Márquez et al.^39^.

### qPCR and RT-qPCR assays

Real-time polymerase chain reaction (qPCR) assays have been widely implemented for estimating total cell count based on DNA gene markers, regardless of their level of metabolic activity^40,41^, while reverse transcription qPCR (RT-qPCR) is a useful method for analyzing the expression of specific genes. RT-qPCR is also used because of its high sensitivity, accuracy, specificity, and rapidity in analyzing the time-specific expression of particular genes, allowing for the detection of low-abundance transcripts^42^. The absolute abundance of total and metabolically active populations of bacteria and *Vibrio* in both target samples were measured by qPCR and RT-qPCR, respectively, using a StepOne Real-Time PCR system (Applied Biosystems, USA). For RT-qPCR, the synthesis of cDNA was performed by reverse transcription of RNA with the aid of SuperScript III Reverse Transcriptase (Invitrogen, Life Technologies Corporation, Carlsbad, CA, EEUU), following the manufacturer’s specifications, in a final volume of 20 µL and using 150–200 ng of total RNA as a template (specific primers described in Table S4) (Sigma Aldrich; St. Louis, MO, USA) and dNTPs (Invitrogen; Carlsbad, USA). In addition, the cDNA quality and concentration were measured using a NanoDrop ND-1000 spectrophotometer (Thermo Scientific Waltham, MA USA). The number of copies (nbc) of 16S rRNA genes (16S rDNA) and 16S rRNA (16S rRNA) were evaluated in each sample using either extracted DNA or cDNA, respectively, as templates with a set of primers previously described (Table S4) based on the increasing fluorescence intensity of the SYBR Green dye during amplification. For the amplification and detection of specific fragments, the iTaq Universal SYBR Green Supermix (Biorad, USA) was used in a final volume of 15 µL for each reaction. All quantitative amplifications were performed in triplicate. The qPCR reaction mixtures contained 1.8 µL of cDNA or DNA, 250 ng of T4 gene 32 (QBiogene, Illkirch, France), 1.2 µL of each primer (10 mM), supplied by Sigma Aldrich (St. Louis, MO, USA), and 1 × SYBR Green Supermix. The amplification and detection conditions are described in Table S5.

To provide absolute quantification of the target microorganisms, standard curves were constructed with the aid of a standard plasmid that contained the inserts of the targeted genes. Amplicons of the 16S rDNA were generated from culture strains of *Pseudomonas putida* NCB957 (quantification of bacteria) and *Vibrio parahaemolyticus* ATCC17802 (quantification of *Vibrio*). The PCR products were cloned with the aid of the pCR2.1-TOPO plasmid vector using the TOPO TA cloning system (Invitrogen, Life Technologies Corporation, Carlsbad, CA, USA), following the manufacturer's protocols. The calibration curves for absolute quantification in the DNA samples (16S rDNA) were generated using serial ten-fold dilutions of linearized plasmid standards, and for absolute quantification in RNA samples (16S rRNA), non-linearized plasmid standards were used as templates for in vitro transcription of the target genes into RNA^39,40,43^. The copy number per ng was calculated as previously described^43^. All calibration curves had a correlation coefficient (r2) of > 0.99 in all assays, and the efficiency of PCR amplification was always between 90 and 110%. Finally, the number of copies of the targeted genes was expressed per gram of tissue sampled, while for water samples these were expressed as the number of copies per mL.

### Statistical analyses

All statistical analyses were performed in R Studio (version 3.6.0)^44^ using the following R packages: maggrittr^45^, ade4^46^, factoextra^47^, vegan^48^, and gplots^49^. Analyses of variance (ANOVA) and multiple-range tests (Student’s-test) were used with a significance level of 95% (*p* < 0.05). Since most of the data sets did not fit the normal distribution, a nonparametric ordination method based on a multiple-comparison test, such as the Kruskall–Wallis test, followed by a Conover–Iman test was conducted for each biological assay, comparing the developmental stage or environment tested (intestine and water) between each shrimp, with a 95% significance level (*p* < 0.05). PCA based on Euclidean distances was used to better explain the analysis of the four sets of biotic data referred to the number of copies per gram of sample of 16S rDNA and 16S rRNA both from total Bacteria and *Vibrio* derived from the quantifications by qPCR/RT-qPCR in water and intestine samples. Moreover, to correlate the influence of the abiotic variables such as NO_2_–N, NH_3_–NH_4_, NO_3_–N, P–PO_4_, DO, salinity, pH, and temperature, with the set of biotic data from the aqueous environment, a PCA was also performed. Differences in gene copy abundance and physical–chemical parameters were evaluated using ANOVA followed by Tukey's honestly significant difference (HSD) tests.

## Supplementary information

Supplementary file1 (DOCX 252 kb)

## References

[1] COSAES. *Comité de Sanidad Acuícola del Estado de Sonora, A. C. Informe final del ciclo 2015*, https://www.cosaes.com/ (2015).

[2] Liu K (2019). Insight into the diversity of antibiotic resistance genes in the intestinal bacteria of shrimp *Penaeus vannamei* by culture-dependent and independent approaches. Ecotoxicol. Environ. Saf..

[3] Fan J (2019). Dynamics of the gut microbiota in developmental stages of *Litopenaeus vannamei* reveal its association with body weight. Sci. Rep..

[4] Tzuc JT, Escalante DR, Rojas Herrera R, Gaxiola Cortés G, Ortiz MLA (2014). Microbiota from *Litopenaeus vannamei*: digestive tract microbial community of Pacific white shrimp (*Litopenaeus vannamei*). SpringerPlus.

[5] Sotomayor MA (2019). Efficacy assessment of commercially available natural products and antibiotics, commonly used for mitigation of pathogenic *Vibrio* outbreaks in Ecuadorian *Penaeus (Litopenaeus) vannamei* hatcheries. PLoS ONE.

[6] Medina-Felix D (2017). Survival of Litopenaeus vannamei shrimp fed on diets supplemented with *Dunaliella* sp. is improved after challenges by *Vibrio parahaemolyticus*. J. Invertebr. Pathol..

[7] Thompson J, Gregory S, Plummer S, Shields RJ, Rowley AF (2010). An in vitro and in vivo assessment of the potential of *Vibrio* spp. as probiotics for the Pacific White shrimp, *Litopenaeus vannamei*. J. Appl. Microbiol..

[8] Almada AA, Tarrant AM (2016). *Vibrio* elicits targeted transcriptional responses from copepod hosts. FEMS Microbiol. Ecol..

[9] Wendling CC, Wegner KM (2015). Adaptation to enemy shifts: rapid resistance evolution to local *Vibrio* spp. in invasive Pacific oysters. Proc. R. Soc. B Biol. Sci..

[10] Roux FL (2015). The emergence of *Vibrio* pathogens in Europe: ecology, evolution, and pathogenesis (Paris, 11–12th March 2015). Front. Microbiol..

[11] Logan, S. L. *et al.* The Vibrio cholerae Type VI Secretion System Can Modulate Host Intestinal Mechanics to Displace Commensal Gut Bacteria. *Biorxiv*, 226472, (2017). 10.1073/pnas.1720133115 PMC5910850 29610339

[12] Welsh RM (2016). Bacterial predation in a marine host-associated microbiome. ISME J..

[13] Luis-Villaseñor IE (2015). Probiotic modulation of the gut bacterial community of juvenile *Litopenaeus vannamei* challenged with *Vibrio parahaemolyticus* CAIM 170. Latin Am. J. Aquat. Res..

[14] Zheng Y (2016). Comparison of cultivable bacterial communities associated with Pacific white shrimp (*Litopenaeus vannamei*) larvae at different health statuses and growth stages. Aquaculture.

[15] Garibay-Valdez E (2019). Biofilm consumption shapes the intestinal microbiota of shrimp (*Penaeus vannamei*). Aquac. Nutr..

[16] Cornejo-Granados F (2017). Microbiome of pacific whiteleg shrimp reveals differential bacterial community composition between wild, aquacultured and AHPND/EMS outbreak conditions. Sci. Rep..

[17] Lemos D, Garcia-Carreno F, Hernández P, Del Toro AN (2002). Ontogenetic variation in digestive proteinase activity, RNA and DNA content of larval and postlarval white shrimp *Litopenaeus schmitti*. Aquaculture.

[18] Xiong J (2017). The underlying ecological processes of gut microbiota among cohabitating retarded, overgrown and normal shrimp. Microb. Ecol..

[19] Schock TB (2013). Evaluation of Pacific white shrimp *(Litopenaeus vannamei)* health during a superintensive aquaculture growout using NMR-based metabolomics. PLoS ONE.

[20] Zhu J (2016). Contrasting ecological processes and functional compositions between intestinal bacterial community in healthy and diseased shrimp. Microb. Ecol..

[21] Zeng S (2017). Composition, diversity and function of intestinal microbiota in pacific white shrimp (*Litopenaeus vannamei*) at different culture stages. PeerJ.

[22] Huang Z, Li X, Wang L, Shao Z (2016). Changes in the intestinal bacterial community during the growth of white shrimp, *Litopenaeus vannamei*. Aquac. Res..

[23] Rungrassamee W (2013). Bacterial population in intestines of the black tiger shrimp (*Penaeus monodon*) under different growth stages. PLoS ONE.

[24] Xiong J (2015). Changes in intestinal bacterial communities are closely associated with shrimp disease severity. Appl. Microbiol. Biotechnol..

[25] Hou D (2018). Comparative analysis of the bacterial community compositions of the shrimp intestine, surrounding water and sediment. J. Appl. Microbiol..

[26] Gainza O, Ramírez C, Ramos AS, Romero J (2018). Intestinal microbiota of white shrimp *Penaeus vannamei* under intensive cultivation conditions in Ecuador. Microb. Ecol..

[27] Huang F, Pan L, Song M, Tian C, Gao S (2018). Microbiota assemblages of water, sediment, and intestine and their associations with environmental factors and shrimp physiological health. Appl. Microbiol. Biotechnol..

[28] Rungrassamee W, Klanchui A, Maibunkaew S, Karoonuthaisiri N (2016). Bacterial dynamics in intestines of the black tiger shrimp and the Pacific white shrimp during *Vibrio harveyi* exposure. J. Invertebr. Pathol..

[29] de Bruijn I, Liu Y, Raaijmakers JM, Wiegertjes GF (2017). Exploring fish microbial communities to mitigate emerging diseases in aquaculture. FEMS Microbiol. Ecol..

[30] Porchas-Cornejo, M. A. *et al.* High-resolution detection of bacterial profile of ocean water, before and after being used by shrimp farms. *Aquacul. Int.*, 1–11 (2017).

[31] Morris JJ (2018). What is the hologenome concept of evolution?. F1000Res.

[32] Garibay-Valdez E (2020). Taxonomic and functional changes in the microbiota of the white shrimp (*Litopenaeus vannamei*) associated with postlarval ontogenetic development. Aquaculture.

[33] Lozupone CA, Knight R (2008). Species divergence and the measurement of microbial diversity. FEMS Microbiol. Rev..

[34] Xiong J, Dai W, Li C (2016). Advances, challenges, and directions in shrimp disease control: the guidelines from an ecological perspective. Appl. Microbiol. Biotechnol..

[35] Xiong, J. *et al. Integrating gut microbiota immaturity and disease-discriminatory taxa to diagnose the initiation and severity of shrimp disease: Gut microbiota diagnoses shrimp disease*. Vol. 19 (2017). 10.1111/1462-2920.13701 28205371

[36] Velázquez-Lizárraga AE (2019). Transcriptomic analysis of Pacific white shrimp (*Litopenaeus vannamei*, Boone 1931) in response to acute hepatopancreatic necrosis disease caused by *Vibrio parahaemolyticus*. PLoS ONE.

[37] Ma X, Sun B, Zhu F (2018). Molecular cloning of Kuruma shrimp *Marsupenaeus japonicus* endonuclease-reverse transcriptase and its positive role in white spot syndrome virus and *Vibrio alginolyticu*s infection. Fish Shellfish Immunol..

[38] Qi C (2017). Transcriptomic and morphological analyses of *Litopenaeus vannamei* intestinal barrier in response to *Vibrio paraheamolyticus* infection reveals immune response signatures and structural disruption. Fish Shellfish Immunol..

[39] Maza-Márquez P (2016). The ratio of metabolically active versus total *Mycolata* populations triggers foaming in a membrane bioreactor. Water Res..

[40] Maza-Márquez P (2019). Abundance of total and metabolically active *Candidatus* Microthrix and fungal populations in three full-scale wastewater treatment plants. Chemosphere.

[41] Kaetzke A, Jentzsch D, Eschrich K (2005). Quantification of *Microthrix parvicella* in activated sludge bacterial communities by real-time PCR. Lett. Appl. Microbiol..

[42] Shakeel M, Rodriguez A, Tahir UB, Jin F (2018). Gene expression studies of reference genes for quantitative real-time PCR: an overview in insects. Biotech. Lett..

[43] Correa-Galeote D, Tortosa G, Bedmar E (2013). Determination of denitrification genes abundance in environmental samples. Metagenomics.

[44] Team, R. C. R: A language and environment for statistical computing. *Vienna, Austria: R Foundation for Statistical Computing. Retrieved from* https://www.R-project.org. Accessed 30 May 2020 (2015).

[45] Bache, S. M. & Wickham, H. magrittr: A Forward-Pipe Operator for R. R package version 1.5. *Vienna, Austria: The R Foundation. Retrieved from* https://CRAN.R-project.org/package=magrittr. Accessed 30 May 2020 (2016).

[46] Chessel, D., Dufour, A. & Thioulouse, J. The ade4 package: Analysis of Ecological Data: Exploratory and Euclidean methods in Environmental sciences. R package version 1.7–13 *R Foundation for Statistical Computing. Retrieved from* https://pbil.univ-lyon1.fr/ADE-4. Accessed 30 May 2020 (2018)

[47] Kassambara, A. & Mundt, F. Factoextra: Extract and visualize the results of multivariate data analyses. R package version 1.0.5. *Retrieved from:* https://www.sthda.com/english/rpkgs/factoextra. Accessed 30 May 2020 (2017).

[48] Oksanen, J. *et al.* Vegan: community ecology package. R package version 2.0-3. *Retrieved from:* https://cran.r-project.org. Accessed 30 May 2020 (2012).

[49] Bonebakker, L. *et al.* R package gplots. Version 3.0. 1. *Retrieved from:* https://github.com/talgalili/gplots. Accessed 30 May 2020 (2012).

