# Supplementary material for: The implication of metabolically active Vibrio spp. in the digestive tract of Litopenaeus vannamei for its post-larval development
Source: Sci Rep. 2020 Jul 10;10:11428. doi: 10.1038/s41598-020-68222-9 (PMC7351783; doi:10.1038/s41598-020-68222-9)
Supplement: Supplementary file 1 — Supplementary file1 (DOCX 252 kb) [file 41598_2020_68222_MOESM1_ESM.docx]

**Supplementary Material**

**Title: The implication of metabolically active *Vibrio* spp. in the digestive tract of *Litopenaeus vannamei* for its post-larval development.**

**Authors:** Estefanía Garibay-Valdez^a^*^†^*, Luis Rafael Martínez-Córdova^b^, Marco A. López-Torres^b^, F. Javier Almendariz-Tapia^c^, Marcel Martínez-Porchas^a^ and Kadiya Calderón^b^*^†^*^*^


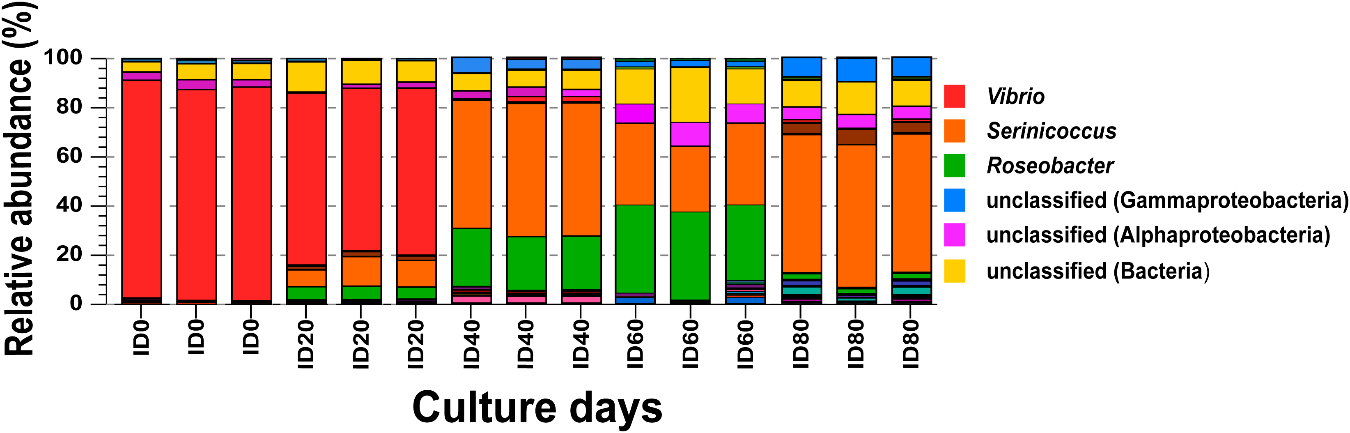


**Figure S1.** Relative abundance (%) of taxonomic changes at genus level detected in gut microbiota of intestine samples (ID) of the white shrimp during its post-larval development.


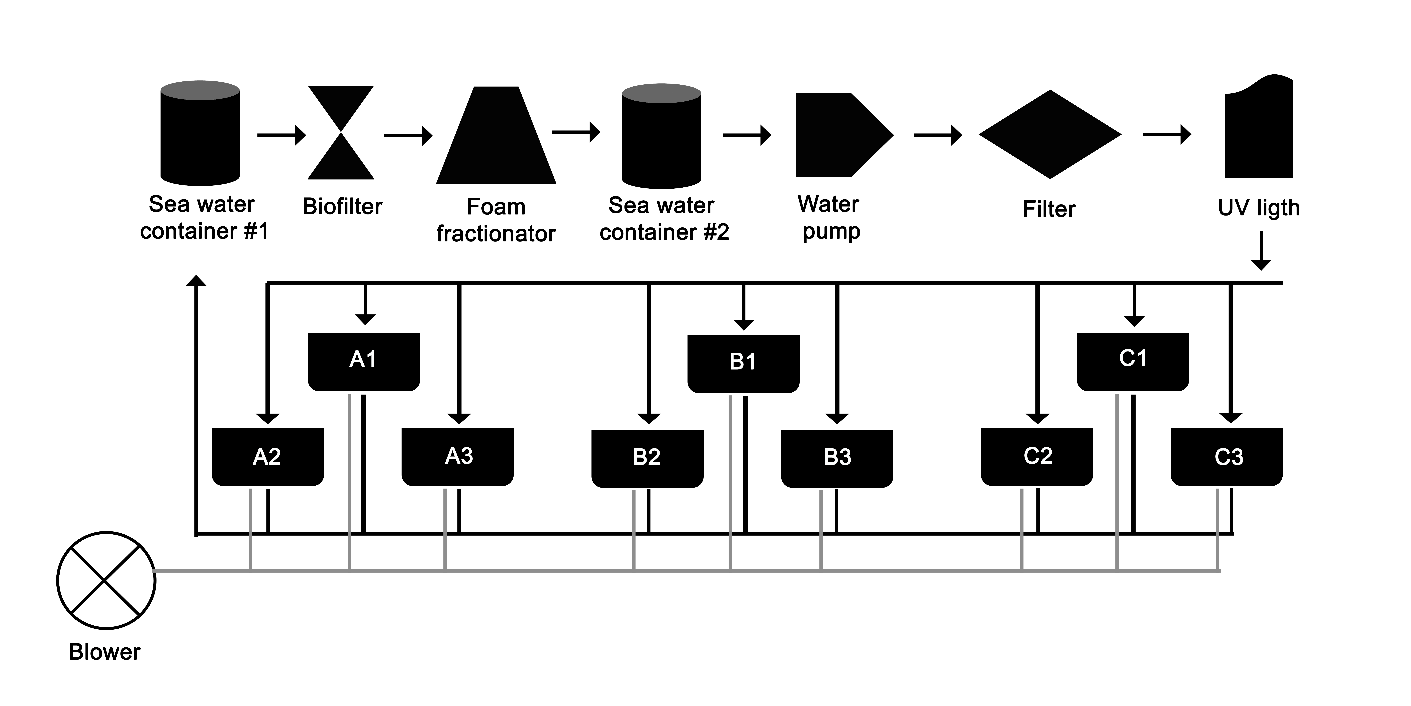


**Fig. S2.** Culture units linked in a recirculation system with filtered sea water

| **Group** | **Gene marker** | **Primer name** | **Sequence (5´- 3´)** | **Reference** |
| --- | --- | --- | --- | --- |
|  |  |  |  |  |
| **Bacteria** | 16S rRNA | 341F  534R | CCTACGGGAGGCAGCAG  ATTACCGCGGCTGCTGG | Maza-Márquez et al. 2016 |
| ***Vibrio* spp.** | 16S rRNA | 567F  680R | GGCGTAAAGCGCATGCAGGT  GAAATTCTACCCCCCTCTACAG | Tall et al. 2012 |

**Table S4.** Primers used for the quantification of total and active populations of bacteria and *Vibrio* spp. in all samples.

| **Sample** | **NH_3_-NH_4_ (mg L^-1^)** | **NO_2_-N**  **(mg L^-1^)** | **NO_3_-N**  **(mg L^-1^)** | **P-PO_4_**  **(mg L^-1^)** | **Temp (°C)** | **Salinity**  **(%)** | **DO**  **(mg L^-1^)** | **pH** |
| --- | --- | --- | --- | --- | --- | --- | --- | --- |
| W0 | 0.39 ± 0.01^(c)^ | 0.01 ± 0.00^(c)^ | 0.0 ± 0^(c)^ | 0.17 ± 0.03^(b)^ | 25.2 ± 0.3 | 37.9 ± 0.7 | 6.87 ± 0.05 | 8.3 ± 0.0 |
| W20 | 5.34 ± 0.16^(a)^ | 0.04 ± 0.00^(c)^ | 0.93 ± 0.49^(c)^ | 1.98 ± 0.20^(a)^ | 25.1 ± 0.6 | 35.4 ± 0.3 | 6.45 ± 0.27 | 8.4 ± 0.1 |
| W40 | 0.93 ± 0.09^(b)^ | 0.11 ± 0.00^(b)^ | 6.50 ± 1.67^(b)^ | 1.66 ± 0.08^(a)^ | 24.7 ± 0.7 | 35.2 ± 0.1 | 6.57 ± 0.25 | 8.1 ± 0.1 |
| W60 | 1.20 ± 0.03^(b)^ | 0.27 ± 0.05^(a)^ | 10.2 ± 1.30^(a)^ | 1.88 ± 0.30^(a)^ | 24.3 ± 1.1 | 35.3 ± 0.4 | 7.07 ± 0.13 | 8.1 ± 0.1 |
| W80 | 0.39 ± 0.01^(c)^ | 0.01 ± 0.00^(c)^ | 0.0 ± 0^(c)^ | 0.17 ± 0.03^(b)^ | 24.9 ± 1.5 | 35.1 ± 0.0 | 7.07 ± 0.13 | 8.0 ± 0.0 |

**Table S5.** Cycling conditions used with RT-qPCR and qPCR for the quantification of the abundance of the total and active population of bacteria and *Vibrio* in shrimp intestine and water samples.
